# Neutrophil Extracellular Traps Exacerbate UVB‐Induced Photodamage in HaCaT Cells and Mouse Skin via CCDC25/MAPK Pathway

**DOI:** 10.1111/acel.70640

**Published:** 2026-07-23

**Authors:** Yang Zou, Junzhi Li, Yuting Peng, Rui Hu, Ruolin Li, Aijun Chen

**Affiliations:** ^1^ Department of Dermatology The First Affiliated Hospital of Chongqing Medical University Chongqing China

## Abstract

The pathogenesis of skin photodamage remains incompletely elucidated. Neutrophil extracellular traps (NETs) have been implicated in exacerbating skin photodamage. However, their precise mechanism requires further investigation. We detected NETs via immunofluorescence in skin tissues from patients with Actinic Dermatosis. In vivo, an acute photodamage mouse model was established by using UVB irradiation. Mice were treated with GSK484 (PAD4 inhibitor) and subjected to RNA‐sequencing to explore the mechanism by which NETs promote inflammation and apoptosis. NETs content, malondialdehyde (MDA) levels, and superoxide dismutase (SOD) activity were assessed. In vitro, immortalized keratinocytes (HaCaT cells) were treated with NETs. Subsequently, cell viability, reactive oxygen species (ROS) levels, inflammatory cytokine release, apoptosis, SOD production, and MDA levels were measured. 25‐pass transmembrane protein (CCDC25) was knocked down to validate its role as a critical receptor for NETs in HaCaT cells. As a result, abundant NETs were present in the skin of Actinic Dermatosis patients. Injection of GSK484 significantly alleviated UVB‐induced skin damage, inflammation, and cell apoptosis in the mouse model. RNA‐sequencing analysis revealed that peptidyl‐arginine deiminase 4 (PAD4) deficiency inhibited UVB‐induced mitogen‐activated protein kinase (MAPK) activation. NETs treatment induced HaCaT cell apoptosis, suppressed cell viability, promoted inflammatory cytokine release, and disrupted redox homeostasis. NETs stimulation increased phospho‐Jun‐N‐terminal kinase (p‐JNK) activity. However, this activity was suppressed and cellular status was restored following CCDC25 knockdown. Collectively, NETs exacerbate skin photodamage by activating the p‐JNK/JNK pathway via CCDC25, leading to HaCaT cell apoptosis, inhibition of cell viability, and the release of inflammatory cytokines.

## Graphical abstract

Neutrophil extracellular traps (NETs) play a pivotal role in UVB‐induced skin photodamage. Mechanistically, NETs bind to the CCDC25 receptor on keratinocytes and subsequently activate the intracellular p‐JNK/JNK signaling pathway, thereby exacerbating skin photodamage.

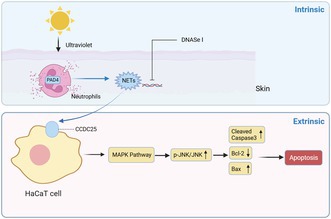

## 1 Introduction

Skin is directly exposed to the external environment and faces risk factors from the environment such as ultraviolet (UV) radiation. UV radiation is categorized by wavelength into UVA (320–400 nm), UVB (280–320 nm), and UVC (100–280 nm). UVA and UVB are the primary wavelengths reaching the Earth's surface and affecting living organisms (McKenzie et al. 2003). Cellular DNA strand damage, oxidative stress, inflammation, and cell apoptosis can be caused by exposure to UVB radiation (Afaq 2011). This is accompanied by the activation of multiple signaling cascades such as mitogen‐activated protein kinase (MAPK) and Nuclear Factor kappa‐B (NF‐κB) pathways in skin cells, exacerbating inflammation and cell apoptosis. Chronically, repeated UV radiation exposure also contributes to skin photoaging and skin carcinogenesis (López‐Camarillo et al. 2012; Manganelli et al. 2025; Muthusamy and Piva 2010; Saewan and Jimtaisong 2015).

Neutrophils play a significant role in UV‐induced skin photodamage. When skin is exposed to UV radiation, the rich vascular network of skin enables rapid neutrophils recruitment and infiltration into the dermis. The recruited neutrophils generate excessive reactive oxygen species (ROS) and proteolytic enzymes under UV radiation, which are released into the extracellular fluid, causing degradation of the extracellular matrix (ECM) and damage to surrounding cells. Additionally, neutrophils produce large quantities of inflammatory cytokines and chemokines, further amplifying neutrophils infiltration and inflammation (Skopelja‐Gardner et al. 2021; Yano et al. 2005). Neutrophil extracellular traps (NETs) are web‐like structures released by neutrophils, composed of proteins and DNA (including both nuclear and mitochondrial DNA). Peptidyl‐arginine deiminase 4 (PAD4), a nuclear citrullinating enzyme, is essential for NET formation in neutrophils. Nuclear PAD4 is activated by calcium ions and ROS, leading to citrullination of histone H3 (Cith3) in NET formation, chromatin decondensation, and subsequent expulsion of the NET meshwork beyond the collapsed nuclear membrane (Mutua and Gershwin 2021; Tan et al. 2021). Targeting PAD4 can therefore inhibit NET generation. While NETs trap and kill pathogens, limiting their invasion and spread as part of innate immunity, excessive NETs formation is implicated in promoting immune‐related diseases and hindering recovery. Consequently, reducing NETs production is emerging as a significant therapeutic strategy to alleviate disease (Fousert et al. 2020). Current research indicates that reducing NET formation mitigates skin photodamage in UVB‐induced photodamaged mouse model (Inaba et al. 2024). However, the precise mechanisms by which NETs contribute to skin photodamage remain unclear.

CCDC25 is a 25‐pass transmembrane protein containing a helical domain. It functions as a receptor for NETs‐DNA on tumor cell surfaces, promoting tumor cell motility and metastasis (Yang et al. 2020). Additionally, CCDC25 is expressed on skin fibroblasts and pulmonary smooth muscle cells, where it participates in NETs‐mediated wound healing and the formation of pulmonary hypertension (Chu et al. 2024; Sun et al. 2024).

In this study, we demonstrate that NETs contribute to UV‐induced photodamage and elucidate that their detrimental effects on both HaCaT cells and mouse skin are mediated through the CCDC25/MAPK pathway.

## 2 Methods

### 2.1 Human Samples

Human skin tissues at the site of the lesions were obtained from 10 patients with Actinic Keratosis or Chronic Actinic Dermatitis from outpatient, and human skin tissues at the site of the non‐exposed areas were obtained from 5 healthy donors from the period of July 2023–December 2024.

### 2.2 Animal Experiments

C57BL/6J mice (20–22 g, male) were purchased from Viton Lihua (Bejing). Mice were all randomly divided into the following groups (six mice/group): control group (WC), GSK484 inhibitor (MCE) group (PC; GSK484 at 4 mg/kg/body weight), UVB irradiation group (WB), and UVB irradiation + GSK484 inhibitor (PB; GSK484 at 4 mg/kg/body weight). The UVB irradiation sources consisted of 2 UVB lamps (Philips, Germany) with a peak intensity of 311 nm. 2 × 2 cm areas were scraped on the dorsal skin of the mice before the UVB irradiation. The mice were exposed to UVB at 430 mj/cm^2^ for two consecutive days. On the third day, the mice were euthanised and the dorsal skin was obtained.

### 2.3 NET Generation

Neutrophils were isolated from freshly collected human peripheral blood and subsequently cultured in RPMI‐1640 medium with 2% fetal bovine serum (Gibco, Grand Island, NY, USA). These cells were maintained at a concentration of 1.5 × 10^6^ cells/mL in Petri dishes measuring 10 cm in diameter. To stimulate the release of NETs, the neutrophils were treated with phorbol 12‐myristate 13‐acetate (PMA, Sigma‐Aldrich, Shanghai, China) at a concentration of 50 nM, 5 μM of ionomycin (MCE, USA) or 1j/cm^2^ UVB irradiation. This incubation continued for 4 h at a temperature of 37°C within an environment containing 5% CO_2_. After incubation, the culture medium was carefully removed without disturbing the NETs that had adhered to the dish's surface. The NETs were then subjected to two distinct PBS rinses and subsequently collected using chilled PBS. After centrifugation for 10 min at 1000 rpm, the supernatant containing the cell‐free NET structures was collected. Finally, the overall NET concentration was determined using a PicoGreen dsDNA Kit (Thermofisher). To visualize NETs formation, neutrophils were stained with the SYTOX green (Beyotime, China) immediately after stimulation and incubation. Images were acquired by fluorescence microscopy, and fluorescence intensity was quantified using Image J software.

### 2.4 Histological Analysis

Specimens were fixed in 4% paraformaldehyde and subsequently embedded in paraffin. 5 μm sections were stained with Hematoxylin and eosin (H&E) staining. Visualization was facilitated through light microscopy. To measure epidermis and dermis thickness, the Image J software was used and measurements were made in three different fields per section.

### 2.5 Cell Culture and Treatment

HaCaT cell was obtained from the American Type Culture Collection (Manassas, VA, USA). Cells were cultured in Dulbecco's modified Eagle's medium (Procell, China) and maintained at 37°C in 5% CO_2_. Subsequently, they were stimulated by PBS, NETs, and NETs + DNase I (Sigma‐Aldrich, China) for 48 h.

### 2.6 siRNA‐Mediated Gene Silencing

Pre‐designed CCDC25 siRNA was purchased from GenePharma. siRNA was transfected using Tuberfect (siRNA‐mate plus, GenePharma) following the manufacturer's protocol. Cells were seeded to be approximately 50%–60% confluent at the time of transfection. The final concentration of siRNA was 10 nM. Cells were incubated at 37°C in 5% CO_2_ for 24 h prior to transfection. The sequences of CCDC25‐siRNA were: S1: 5′‐CCUAGCAGCAGAGAAAGAATT‐3′ and 5′‐UUCUUUCUCUGCUGCUAGGTT‐3′; S2: 5′‐CACAGGCAGAAGGAUGUAATT‐3′ and 5′‐UUACAUCCUUCUGCCUGUGTT‐3′; S3: 5′‐CAGCAGCGUUAAUUCAUCUTT‐3′ and 5′‐AGAUGAAUUAACGCUGCUGTT‐3′. Then, cells were washed with ice‐cold 1X PBS after transfection for 24 h and they were treated with NETs subsequently.

### 2.7 Immunofluorescence

Following the removal of paraffin and hydration, the tissue specimens were immersed in Citrate antigen retrieval buffer, and then subsequently treated with a 0.1% Triton‐X 100 solution for permeabilization. Then 5% BSA was used for 30 min at 37°C to minimize nonspecific binding. The following primary antibodies were used: CitH3 (Cell Signaling Technology, USA), NE (Abcam, USA). The recombinant secondary antibody (Proteintech, China) was used. Additionally, 4,6‐diamidino‐2‐phenylindole (DAPI, Beyotime, China) was used to stain the cell nuclei. Images were captured via ortho‐fluorescence microscopy, and the area of positive staining and the number of positive cells were analyzed via Image J.

### 2.8 Reactive Oxygen Species Assay

The ROS Assay Kit (Beyotime, China) was used to detect the intracellular ROS levels of HaCaT cells after the stimulation of NETs. The experimental procedure was performed following the manufacturer's protocol. Images were captured via ortho‐fluorescence microscopy, and images were analyzed via Image J.

### 2.9 Lipid Peroxidation MDA Assay and Total Superoxide Dismutase Assay

MDA Assay Kit (Beyotime, China) and SOD Assay Kit (Beyotime, China) were used to detect the intracellular MDA and SOD levels of HaCaT cells after the stimulation of NETs and the MDA, SOD levels of skin samples of mice. The experimental procedures were performed according to the manufacturer's protocol, and the absorbance at 532 nm (MDA)/450 nm (SOD) was detected by a Microplate reader (Thermofisher).

### 2.10 Flow Cytometry

HaCaT cells' apoptosis was assessed by the Annexin V‐FITC Apoptosis Assay (Beyotime, China). After all the treatments, the cells were harvested and stained with Annexin V‐FITC and propidium iodide (PI) for the identification of apoptotic cells. The staining protocol was carried out according to the manufacturer's recommendations. The flow cytometric analysis was conducted using a flow cytometer.

### 2.11 Cleaved Caspase‐3 Immunofluorescence and TUNEL Co‐Staining

Paraffin‐embedded skin sections were deparaffinized, rehydrated, and treated with proteinase K for antigen retrieval. After post‐fixation in 4% PFA, Colorimetric TUNEL Apoptosis Assay Kit (Beyotime, China) was used to detect the extent of apoptosis in skin samples according to the manufacturer's instruction. Sections were then blocked with 3% BSA and incubated overnight at 4°C with anti‐Cleaved Caspase‐3 antibody (CST, USA). After washing, the recombinant secondary antibody (Proteintech, China) was applied for 1 h at room temperature. Nuclei were counterstained with DAPI. Images were acquired under a fluorescence microscope.

### 2.12 Cell Counting Kit‐8 Assay

HaCaT cells were dispensed into a 96‐well plate, with each well receiving 5000 cells. After a 24‐h window for the cells to adhere and proliferate, they were then incubated in different concentrations of NETs for another 48 h. CCK8 Kit (MCE, USA) was used to assess cell viability. 10 μL of CCK‐8 solution was added to each well, and the cells were incubated for an additional 1 h. The optical density at 490 nm was measured using a plate reader.

### 2.13 Western Blotting

Skin sample was ground by magnetic beads, and proteins were extracted using RIPA buffer (Beyotime, China). Cells were harvested and lysed on ice with RIPA buffer. BCA Protein Assay Kit (Thermofisher) was used to determine protein concentration. The protein samples were analyzed on 10% SDS‐PAGE and transferred onto a polyvinylidene fluoride (PVDF) membrane. The membranes were blocked with 5% non‐fat milk for 1 h at room temperature, washed three times in TBST, and incubated with the primary antibody overnight at 4°C. The membranes were washed three times with TBST and incubated with horseradish peroxidase secondary antibodies (ABclonal, Wuhan, China) for 1 h at room temperature. The bands were detected using Western ECL Blotting Substrates and an imaging system from Bio‐Rad (Shanghai, China) according to the manufacturer's protocols. The following primary antibodies were used: JNK, phospho‐JNK (p‐JNK), p38, phospho‐p38 (p‐p38), ERK, phospho‐ERK (p‐ERK), BAX, BCL‐2, Cleaved Caspase‐3, CCDC25, and β‐actin. ImageJ software was used to quantify the densities of specific bands.

### 2.14 Real‐Time Reverse Transcription PCR

Total RNA was extracted from cell lines or mice using RNAiso Plus (Takara), and reverse‐transcribed to cDNA using the HiScript Q RT SuperMix from the qPCR kit (Yeason). RT‐PCR was performed using specific primers, SYBR Green qPCR Master Mix (Selleck), and detected using the CFX96TM real‐time PCR Detection System (Bio‐Rad, Shanghai, China). The primers used for RT‐PCR are shown in Table S1.

### 2.15 RNA‐Sequencing

Total RNA was extracted from photodamaged and non‐photodamaged skin samples using RNAiso Plus (Takara). Subsequently, RNA samples were detected using the Nanodrop ND‐2000 system (ThermoFisher) based on the A260/A280 absorption ratio. Then, RNA library sequencing was performed using the DNBSEQ‐T7 instrument (MGI, China). Differentially expressed genes (DEGs) were identified using the DESeq2 R package, the screening criteria were *p* < 0.05 and |log2 fold change| > 1.

### 2.16 Adeno‐Associated Virus (AAV) Vector Construction

Adeno‐associated virus (AAVDJ) vectors for Mouse CCDC25 knockdown and scrambled control (GFP) were constructed, amplified, and purified by Tsingke Biotechnology (Beijing, China). Each AAV vector achieved a final concentration of 1 × 10^13^ vgs/ml. A 20 μL volume of the AAV diluted solution for local delivery was injected intracutaneously into the dorsal skin of the experimental mice. Subsequently, UVB was used to establish a mouse model of skin photodamage.

### 2.17 Statistical Analyses

All experiments were performed in triplicate. The data were analyzed using GraphPad Prism 9.3 (GraphPad Software, La Jolla, CA). The values were expressed as mean ± standard deviation, computed for each group, and analyzed using one‐way analysis of variance (ANOVA) or the Student's *t*‐test. A *p*‐value of < 0.05 was considered to indicate a statistically significant difference.

## 3 Results

### 3.1 Increased NETs Levels in Actinic Dermatitis Skin Lesions

**FIGURE 1:**
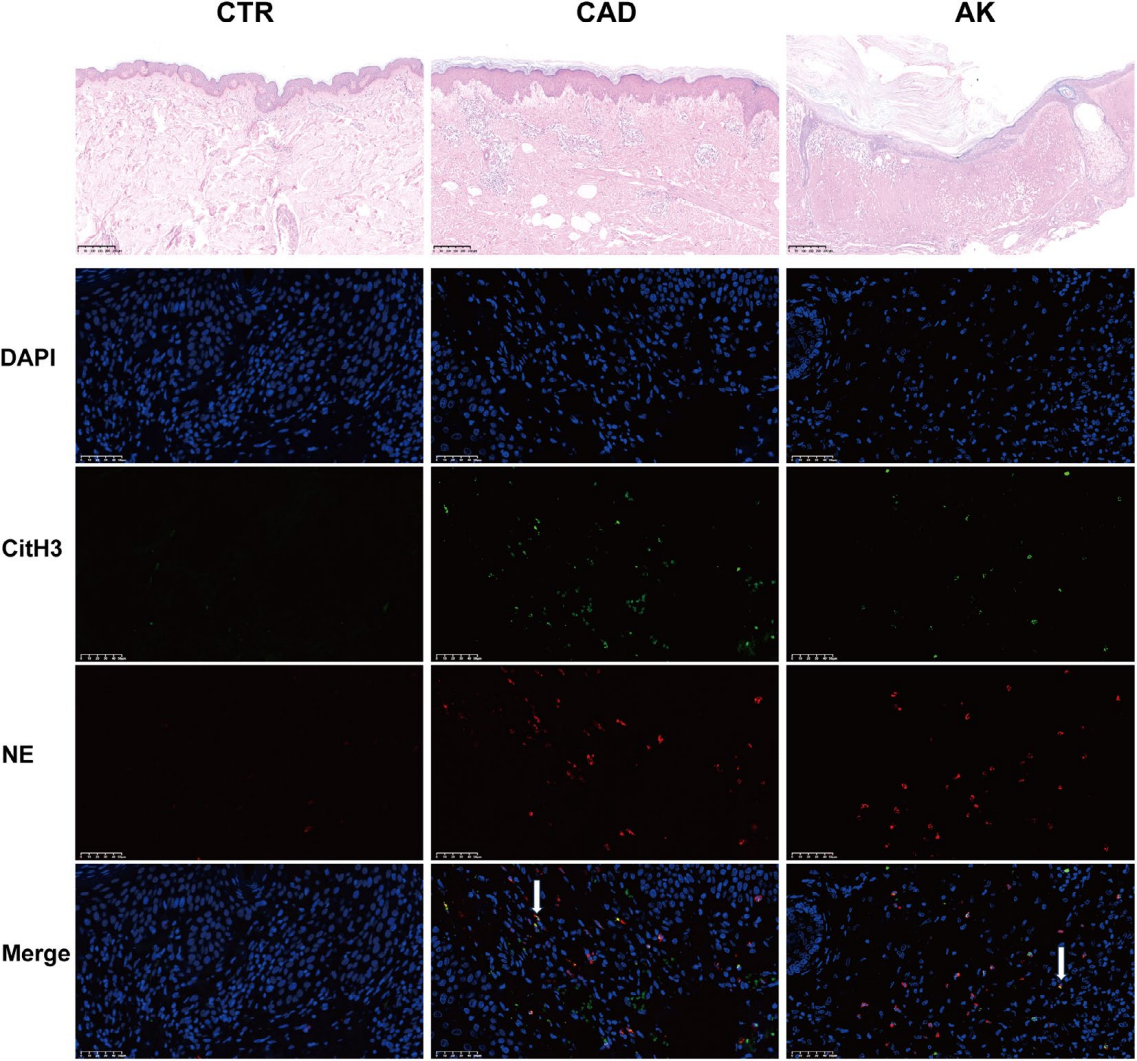
Immunofluorescence visualization of NETs expression in skin samples of Patients with Chronic Actinic Dermatitis or Actinic Keratosis. Immunofluorescence staining of NET (marked by anti‐CitH3 and anti‐NE antibodies, Green: CitH3, Red: NE, Blue: DAPI) structures in the skin of Chronic Actinic Dermatitis or Actinic Keratosis patients and controls. Scale bars, 50 μm; *n* = 5 per group. The same tissue sections were also stained with H&E. Scale bars, 250 μm. Each experimental procedure was independently replicated three times, consistently yielding similar results.

Compared to healthy human skin, H&E staining revealed epidermal thickening in patients with AK or CAD. CitH3 and NE co‐localization were used in IF staining to mark NETs deposition. It revealed increased NETs deposition in the skin lesions of AK and CAD patients (Figure 1). These findings indicate that UV exposure can induce neutrophil infiltration into the skin and substantial NETs formation.

### 3.2 NETs Inhibition Alleviates UVB‐Induced Skin Photodamage in Mice

**FIGURE 2:**
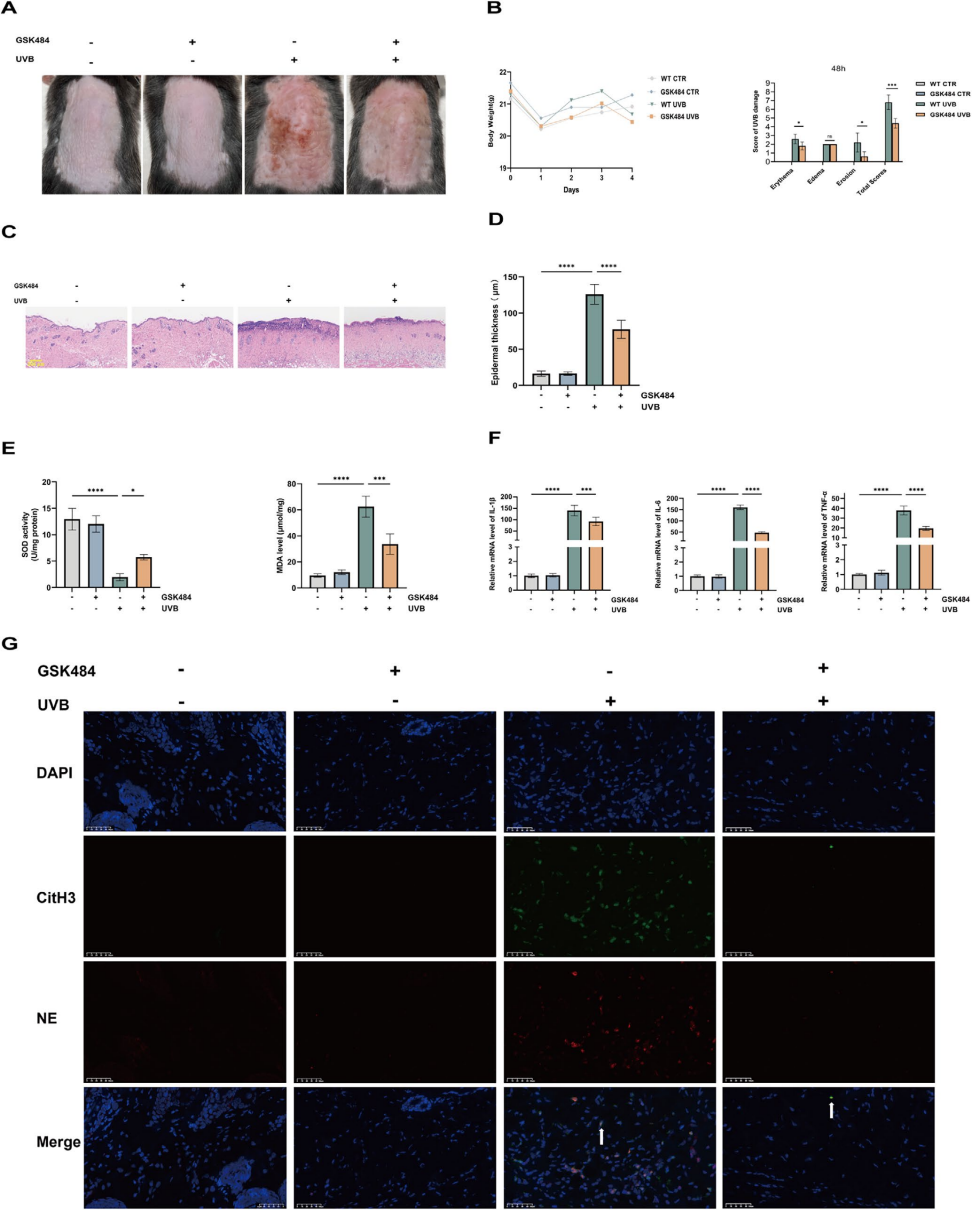
GSK484 inhibits NETs generation and alleviates UVB‐induced skin photodamage in mice. (A) GSK484 ameliorates the skin appearance in UVB‐exposed mice. (B) Changes in body weight and dermatitis score during the experimental period. (C) H&E staining of skin sections across experimental groups. Scale bar: 200 μm.(D) Quantification of the thickness of skin in the H&E staining. (E) SOD activity and MDA content in mouse skin samples. (F) Relative mRNA levels of IL‐1β, IL‐6, and TNF‐α in mouse skin samples. (G) Immunofluorescence analysis of NETs formation (Green: CitH3, Red: NE, Blue: DAPI) in mouse skin samples with or without GSK484 treatment. Scale bar: 50 μm. *n* = 6 per group. The values are presented as the mean ± SD. *p < 0.05, **p < 0.01, ***p < 0.001. Each experimental procedure was independently replicated three times, each yielding similar results.

PAD4 is a key enzyme promoting NETs formation, and inhibiting PAD4 suppressed NETs generation (Mutua and Gershwin 2021). We established a mouse skin photodamaged model by irradiating the shaved dorsal skin of C57BL/6J mice with UVB. A subset of mice was treated with GSK484, and the extent of skin photodamage was assessed by a scale. UVB‐irradiated mice exhibited acute photodamage manifestations such as erythema and exudation (Figure 2A). However, mice that received intraperitoneal GSK484 injections during irradiation showed reduced total skin lesion scores (Figure 2B). We obtained results similar to Inaba et al. (2024), indicating that inhibiting NETs formation ameliorates UVB‐induced skin photodamage. H&E staining and quantitative analysis revealed that GSK484 treatment ameliorated UVB‐induced epidermal thickening (Figure 2C,D). What's more, we assessed redox imbalance in the photodamaged model by measuring MDA and SOD levels in skin tissue homogenates. The results showed increased MDA levels in the untreated photodamaged mice group, which were reduced in GSK484‐treated photodamaged mice. SOD levels were increased in GSK484‐treated photodamaged mice compared to untreated photodamaged mice (Figure 2E). Furthermore, the increase in the expression of inflammation‐related markers, including TNF‐α, IL‐1β and IL‐6, at the mRNA level observed in photodamaged mice was significantly attenuated after NETs suppression (Figure 2F). IF staining revealed abundant NETs deposition in the skin lesions of UVB‐induced mice. GSK484 treatment suppressed NETs generation (Figure 2G). Furthermore, we established a UVB‐induced skin photodamage model using PAD4‐knockout mice and found that PAD4 deficiency significantly alleviated the photodamage (Figure S2). These results demonstrate that NETs produced by neutrophils can exacerbate UVB‐induced skin photodamage. Therefore, inhibiting NETs formation can ameliorate photodamage.

### 3.3 GSK484 Ameliorates Skin Cell Apoptosis in Photodamaged Mice

**FIGURE 3:**
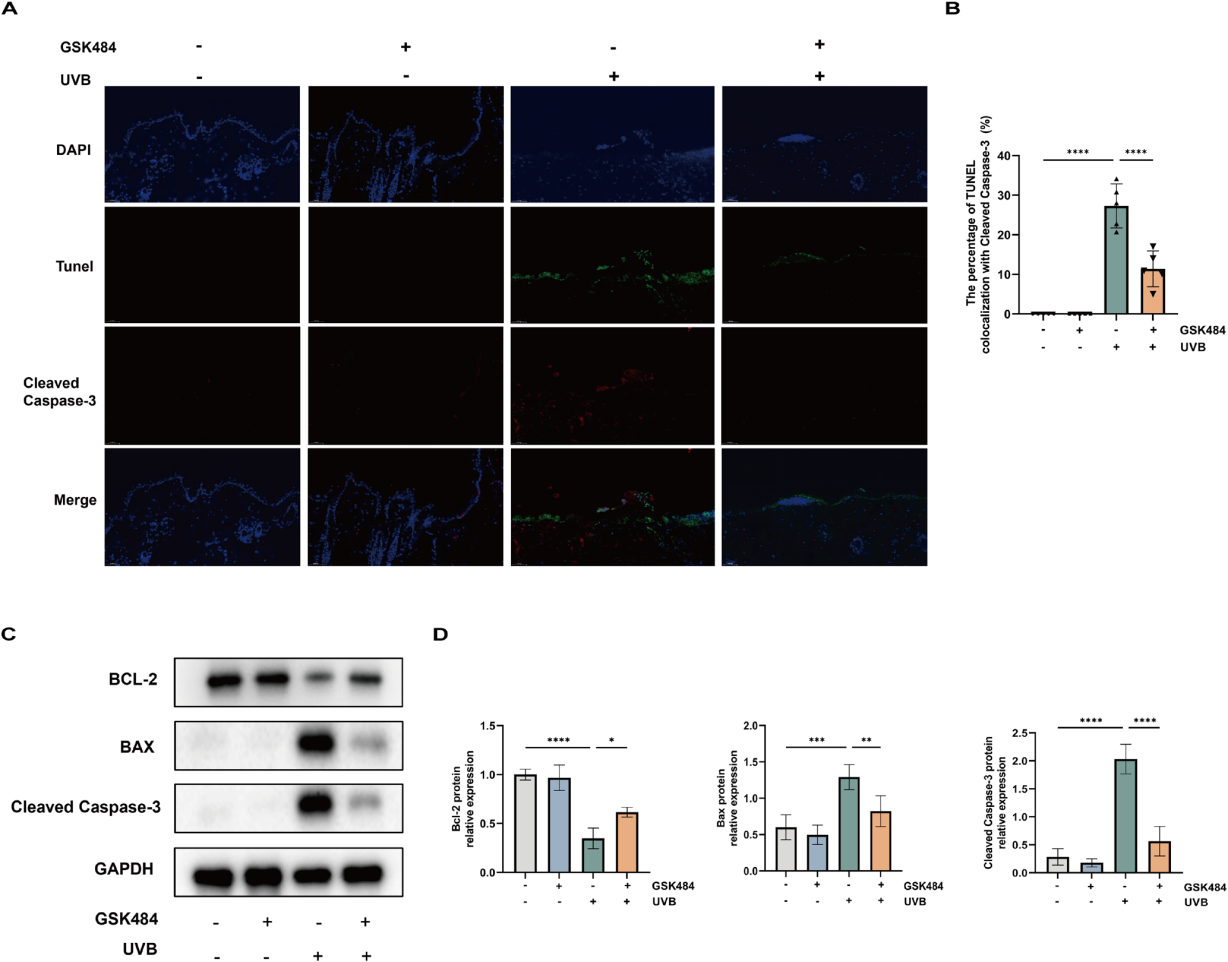
GSK484 ameliorates skin cell apoptosis in photodamaged mice. (A) Co‐staining of TUNEL and Cleaved Caspase‐3 in representative mice of each experimental group. Scale bar: 50 μm. (B) Quantitative analysis of TUNEL and Cleaved Caspase‐3 co‐staining across experimental groups. (C) Protein levels of Cleaved Caspase‐3, BCL‐2, and BAX in skin samples detected by Western Blotting. (D) Quantification of results shown in panel (C). The values are presented as the mean ± SD. *p < 0.05, **p < 0.01, ***p < 0.001. Each experimental procedure was independently replicated three times, each yielding similar results.

Co‐staining of TUNEL and Cleaved Caspase‐3 was used to assess apoptosis in the mouse model. Substantial research supports that keratinocytes or fibroblasts undergo apoptosis in UV‐induced skin photodamaged models, and inhibiting apoptosis can improve the extent of skin damage (Lee et al. 2013; Pustisek and Situm 2011). Our study found that GSK484 treatment significantly reduced the degree of apoptosis. Co‐staining of TUNEL and Cleaved Caspase‐3 results revealed a marked decrease in the co‐staining positivity rate following NETs inhibition (Figure 3A,B). Additionally, we measured markers of apoptosis including BCL‐2, BAX, and Cleaved Caspase‐3 by Western blotting (WB) (Figure 3C,D). These results also indicated that inhibiting NETs formation ameliorates UVB‐induced apoptosis in photodamaged skin cells.

### 3.4 GSK484 Alleviates UVB‐Induced Skin Photodamage via the MAPK Pathway

**FIGURE 4:**
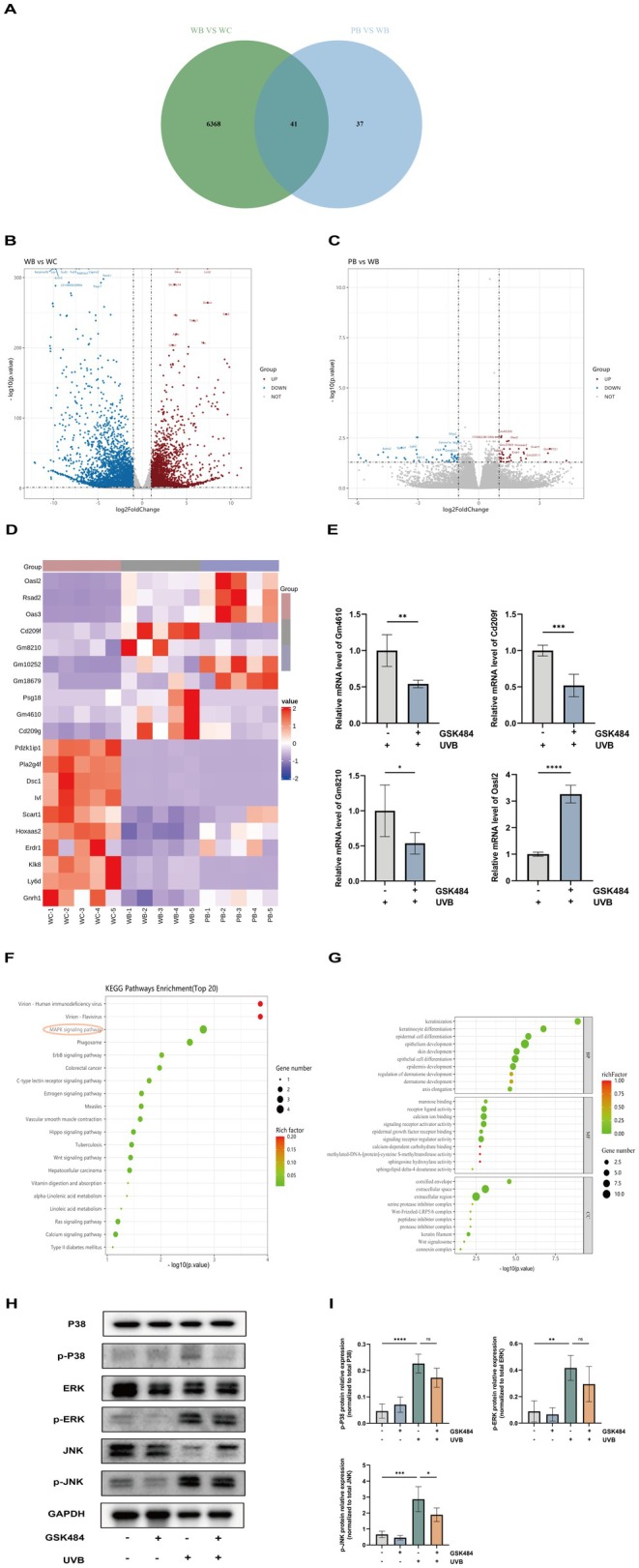
GSK484 alleviates UVB‐induced skin photodamage by inhibiting the MAPK pathway. (A) Venn diagram showing overlapping differentially expressed genes (DEGs) between WB vs. WC and PB vs. WB. Groups: WB (UVB‐irradiated), WC (control), PB (UVB + GSK484 treatment). (B, C) Volcano plots of DEGs in WB vs. WC (B) and PB vs. WB (C). Blue: Downregulated genes; red: Upregulated genes. (D) Heatmaps displaying up or down regulated genes in the WC vs. WB and WB vs. PB comparisons (E). Representative genes were validated by qPCR. (F) Bubble plot of KEGG enrichment analysis for downregulated DEGs in PB vs. WB. (G) Bubble plot of GO enrichment analysis for downregulated DEGs in PB vs. WB. (H) Protein levels detected by Western Blotting of P38, p‐P38, ERK, p‐ERK, JNK, and p‐JNK in skin samples. (I) Quantification of results shown in panel (H). The values are presented as the mean ± SD. *p < 0.05, **p < 0.01, ***p < 0.001. Each experimental procedure was independently replicated three times, each yielding similar results.

To understand the mechanism of PAD4 in the UVB‐induced skin photodamaged model, we performed RNA‐sequencing on the WC, WB, and PB groups. 6409 DEGs showed significant expression differences between the WC and WB groups, 78 DEGs among the PB and WB groups, and 41 DEGs significantly expressed across all three groups (WB, WC, and PB) (Figure 4A). Volcano plots displayed the significantly DEGs, with blue indicating downregulated genes and red indicating upregulated genes. The top 10 most significantly up or down genes are labeled with their gene symbols (Figure 4B,C). Heatmaps displayed up or downregulated genes in the WC vs. WB and WB vs. PB comparisons. PCR validated the sequencing results, yielding consistent findings (Figure 4D,E).

Kyoto Encyclopedia of Genes and Genomes (KEGG) enrichment analysis was performed on the significantly downregulated genes in the PB vs. WB comparison and it revealed the top 20 enriched pathways, including the MAPK and Hippo signaling pathways (Figure 4F). The MAPK pathway is a core signaling network regulating inflammation and immune responses, consisting of three main branches: ERK, p38, and JNK (Muthusamy and Piva 2010). Studies have reported MAPK involvement in NETs‐mediated apoptosis (Hu, Hua, et al. 2023; Hu, Li, et al. 2023). Consistent with the sequencing data, our Western blotting results demonstrated that NETs activate the MAPK pathway, in which the p‐JNK/JNK pathway is activated. However, suppressing NETs generation via intraperitoneal GSK484 injection suppressed p‐JNK/JNK pathway activation (Figure 4H,I). Gene Ontology (GO) analysis of downregulated genes in the WB vs. PB group indicated abnormal keratinocyte proliferation and impaired differentiation function in UVB‐induced photodamage (Figure 4G). These impairments were ameliorated following PAD4 inhibition, which corresponds to reduced NETs levels.

These results demonstrated that UVB irradiation induced NETs generation, which in turn activates the p‐JNK/JNK signaling pathway within the MAPK cascade. This activation promotes apoptosis and exacerbates skin photodamage. Inhibiting NETs generation significantly ameliorates UVB‐induced skin photodamage.

### 3.5 DNase I Prevents NETs‐Induced Suppression of HaCaT Cell Proliferation, Exacerbation of Inflammation, and Redox Imbalance

**FIGURE 5:**
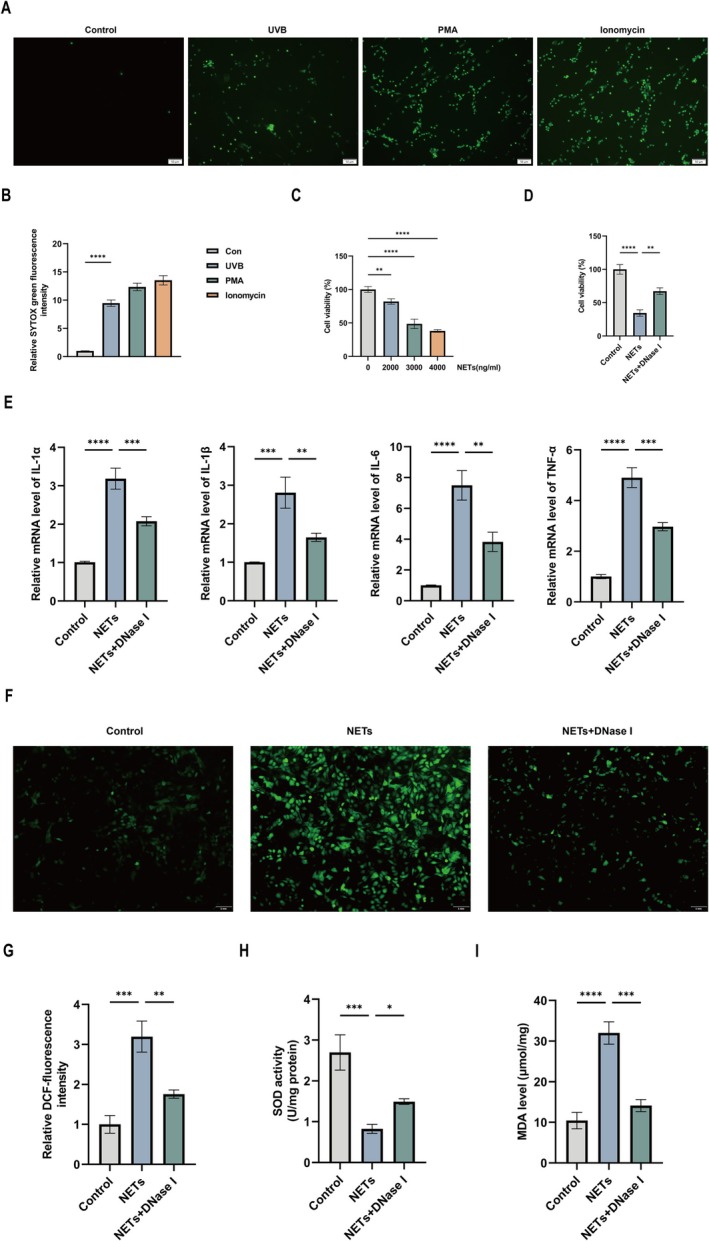
DNase I prevents NETs‐induced suppression of HaCaT cell proliferation, exacerbated inflammation, and redox imbalance. (A,B) Fluorescence microscopy and quantitative analysis demonstrated that NETs formation was induced by the stimulation with PMA, ionomycin, and UVB. (C,D) Cell viability was assessed by CCK‐8 reagent after NETs intervention and after NETs disruption by DNase I. (E) mRNA levels of IL‐1α, IL‐1β, TNF‐α, and IL‐6 following NETs disruption by DNase I (qPCR). (F,G) Intracellular ROS levels detected by fluorescent probes after NETs disruption by DNase I. (H,I) MDA content and SOD activity after NETs disruption by DNase I. The values are presented as the mean ± SD. *p < 0.05, **p < 0.01, ***p < 0.001. Each experimental procedure was independently replicated three times, each yielding similar results.

In animal models, we demonstrated that NETs inhibition ameliorates UVB‐induced skin photodamage, reduces apoptosis, alleviates skin inflammation, and mitigates oxidative stress. To directly observe the effect of NETs on HaCaT cells, human peripheral blood neutrophils were first isolated and stimulated with PMA, ionomycin, or UVB to induce NETs formation. SYTOX green staining revealed that UVB induced robust NETs release (Figure 5A,B). Accordingly, neutrophils were stimulated with UVB to generate NETs, which were subsequently used to treat HaCaT cells. Previous studies indicate that low concentrations of NETs can induce keratinocyte proliferation, while high concentrations exert cytotoxic effects on HaCaT cells (Tonello et al. 2017). After extracting and quantifying NETs in vitro, we treated HaCaT cells and found that there was a most significant impact on suppressing cell proliferation activity with 4000 ng/mL NETs (Figure 5C). Therefore, this concentration was used for the subsequent studies.

Pre‐mixing NETs with DNase I for 30 min allowed DNase I to hydrolyze the DNA backbone, directly disrupting NETs structure (Demkow 2023) and restoring HaCaT cell proliferative activity (Figure 5D). Furthermore, we observed that NETs treatment promoted increased inflammatory cytokine formation in HaCaT cells, activating inflammatory responses within the keratinocytes (Figure 5E). NETs also induced redox imbalance in keratinocytes, manifested by increased ROS levels, elevated lipid peroxidation, and decreased activity of the antioxidant enzyme SOD (Figure 5F–I). These results indicated that, similar to in vivo findings, NETs formation leads to an inflammatory state and redox imbalance in keratinocytes.

### 3.6 DNase I Inhibits NETs‐Induced HaCaT Cell Apoptosis by Blocking P‐JNK/JNK Pathway Activation

**FIGURE 6:**
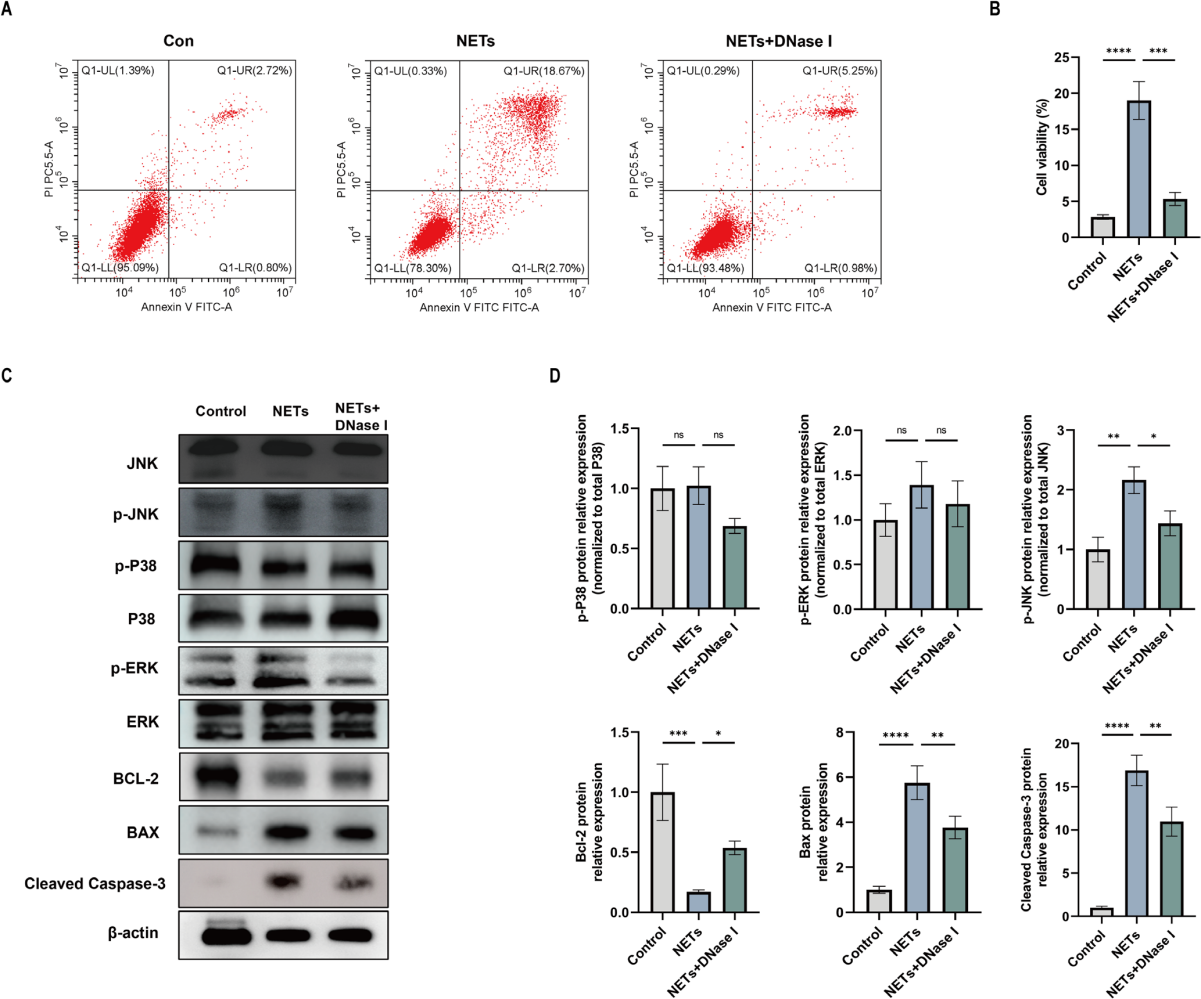
DNase I Inhibits NETs‐induced HaCaT cell apoptosis by blocking p‐JNK/JNK pathway activation. (A‐B) Flow cytometry analysis of apoptosis levels following NETs disruption by DNase I. (C) Protein levels of Cleaved Caspase‐3, BCL‐2, BAX, P38, p‐P38, ERK, p‐ERK, JNK, and p‐JNK detected by Western Blotting in HaCaT cells. (D) Quantification of results shown in panel (C). The values are presented as the mean ± SD. *p < 0.05, **p < 0.01, ***p < 0.001. Each experimental procedure was independently replicated three times, each yielding similar results.

Previous studies indicate that NETs can induce apoptosis in neuronal cells, and NETs inhibition ameliorates apoptosis (Shi et al. 2023). However, whether NETs induce apoptosis in keratinocytes still remains unclear. We treated keratinocytes with high concentrations of NETs and assessed apoptosis using flow cytometry. The results revealed that NETs induced apoptosis in HaCaT cells. However, treatment with DNase I, which disrupts NETs structure, rescued the cells from apoptosis (Figure 6A,B). As previously mentioned, RNA‐sequencing of UVB‐induced photodamaged mouse skin showed that NETs inhibition reduced p‐JNK/JNK pathway activity, suggesting NETs may act via this pathway. To investigate the specific mechanism of NETs‐induced apoptosis in keratinocytes, we intervened with keratinocytes using NETs which we extracted in vitro, either pre‐incubated or not with DNase I. The results revealed that the NETs treatment group, compared to the control and DNase I + NETs groups, specifically activated the p‐JNK/JNK pathway within the MAPK cascade, without significant activation of p‐p38/p38 or p‐ERK/ERK. Concurrently, the pro‐apoptotic proteins BAX and Cleaved Caspase‐3 were significantly upregulated, while the anti‐apoptotic protein BCL‐2 was downregulated (Figure 6C,D). These findings indicated that NETs promote keratinocyte apoptosis specifically through activation of the p‐JNK/JNK signaling pathway.

### 3.7 Knockdown of CCDC25 Impedes NETs‐Induced P‐JNK/JNK Pathway Activation and HaCaT Cell Apoptosis

**FIGURE 7:**
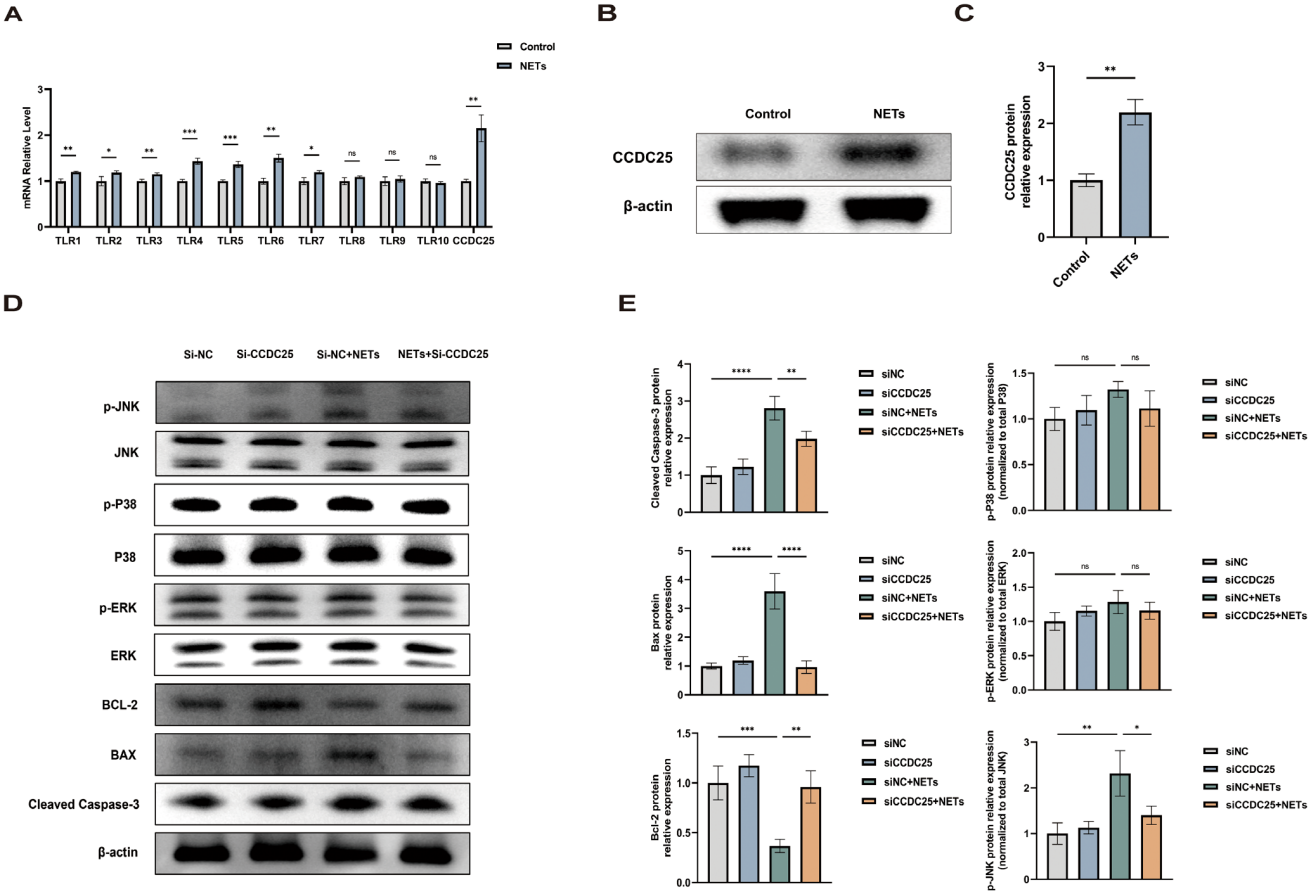
CCDC25 knockdown inhibits NETs‐induced p‐JNK/JNK pathway activation and HaCaT cell apoptosis. (A) Relative mRNA levels of TLRs and CCDC25 in HaCaT cells following NETs stimulation (qPCR). (B,C) CCDC25 protein levels in HaCaT cells after NETs stimulation detected by Western blotting. (D,E) Protein levels of Cleaved Caspase‐3, BCL‐2, BAX, P38, p‐P38, ERK, p‐ERK, JNK, and p‐JNK in CCDC25‐knockdown HaCaT cells following NETs stimulation detected by Western blotting. The values are presented as the mean ± SD. *p < 0.05, **p < 0.01, ***p < 0.001. Each experimental procedure was independently replicated three times, each yielding similar results.

In the in vitro models, NETs can function through Toll‐like receptors (TLRs) on cells (Schneider et al. 2021; Yang et al. 2023). TLR4, as a receptor on keratinocytes, participates in NETs‐mediated inflammation and immune responses (Shao et al. 2019). CCDC25, a coiled‐coil domain‐containing 25‐pass transmembrane protein, exerts NETs functions by binding to DNA within NETs (Yang et al. 2020). To investigate which receptor plays the primary role during NETs intervention, we extracted cellular RNA for PCR after treating keratinocytes with NETs. The results revealed increased expression of some TLRs and CCDC25 under NETs stimulation, with CCDC25 exhibiting the most notable increase (Figure 7A). Consistent with the mRNA levels, WB results also confirmed elevated CCDC25 protein levels in NETs‐treated keratinocytes (Figure 7B,C); Similarly, in fibroblasts, NETs function depended on CCDC25 (Chu et al. 2024); In our UVB‐induced mouse skin photodamaged model, the UVB‐treated group showed the highest CCDC25 expression. CCDC25 expression decreased following NETs inhibition, further indicating cellular upregulation of CCDC25 in response to NETs stimulation (Figure S1).

To determine whether NETs influence p‐JNK/JNK pathway activation specifically through CCDC25 or not, CCDC25 was knocked down in keratinocytes using small interfering RNA (siRNA). CCDC25 knockdown efficiency is shown in Figure S3. One successfully knockdown sequence was selected for the subsequent experiments. WB results demonstrated that after CCDC25 knockdown, NETs‐stimulated p‐JNK/JNK pathway activity returned to baseline levels, while p‐p38/p38 and p‐ERK/ERK activities remained unaffected. Correspondingly, the levels of NETs‐stimulated apoptosis‐related proteins also normalized after CCDC25 knockdown (Figure 7D,E) and improved inflammation levels (Figure S4). These results indicated that CCDC25 plays a critical role in NETs‐induced activation of the p‐JNK/JNK pathway and apoptosis in HaCaT cells.

### 3.8 CCDC25 Knockdown via Adeno‐Associated Virus Injection Alleviates UVB‐Induced Skin Photodamage by Inhibiting the p‐JNK/JNK Pathway

**FIGURE 8:**
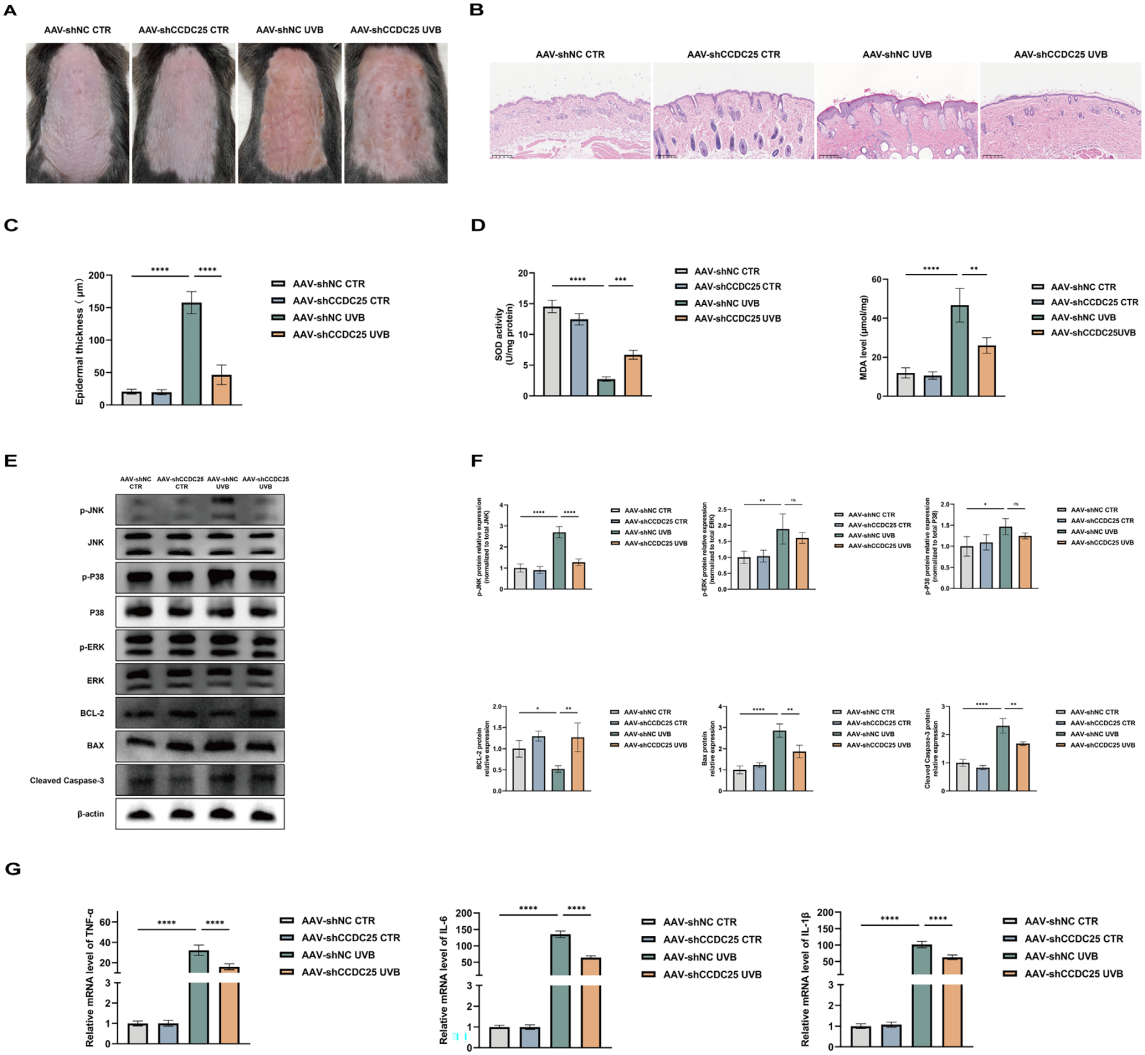
CCDC25 knockdown via adeno‐associated virus injection into the dorsal skin of mice alleviates UVB‐induced skin photodamage by inhibiting the p‐JNK/JNK signaling pathway. (A) CCDC25 knockdown ameliorates the skin appearance in UVB‐exposed mice. (B) H&E staining of skin sections across experimental groups. Scale bar: 200 μm. (C) Quantification of the thickness of skin in the H&E staining. (D) SOD activity and MDA content in mouse skin samples. (E) Protein levels of Cleaved Caspase‐3, BCL‐2, BAX, P38, p‐P38, ERK, p‐ERK, JNK, and p‐JNK in skin samples detected by Western Blotting. (F) Quantification of results shown in panel (E). (G) Relative mRNA levels of IL‐1β, IL‐6, and TNF‐α in mouse skin samples. The values are presented as the mean ± SD. *p < 0.05, **p < 0.01, ***p < 0.001. Each experimental procedure was independently replicated three times, each yielding similar results.

To elucidate the role of CCDC25 in NETs‐mediated promotion of UVB‐induced skin photodamage, we used AAV‐shCCDC25 to knock down CCDC25 expression and generated a UVB‐induced mouse model of skin photodamage. We injected AAV‐shCCDC25 and AAV‐shNC into the dorsal skin of mice. After 3 weeks, CCDC25 expression was significantly reduced in AAV‐shCCDC25‐treated mice compared to AAV‐shNC‐treated mice (Figure S5). Subsequently, we constructed a UVB‐induced skin photodamage model. UVB‐induced photodamage was significantly less severe in AAV‐shCCDC25‐treated mice compared to AAV‐shNC‐treated mice, with reduced skin erythema and exudation (Figure 8A). H&E staining and quantitative analysis revealed that AAV‐shCCDC25 treatment ameliorated UVB‐induced epidermal thickening (Figure 8B,C). Additionally, we found that increased MDA levels in AAV‐shNC‐treated photodamaged mice group, which were reduced in AAV‐shCCDC25‐treated photodamaged mice. SOD level was increased in AAV‐shCCDC25‐treated photodamaged mice compared to AAV‐shNC‐treated photodamaged mice (Figure 8D). WB results demonstrated that the p‐JNK/JNK pathway and apoptosis‐related proteins were upregulated in AAV‐shNC‐treated photodamaged mice, but downregulated in the AAV‐shCCDC25‐treated mice (Figure 8E,F). Furthermore, CCDC25 knockdown significantly attenuated the upregulation of TNF‐α, IL‐1β, and IL‐6 mRNA observed in photodamaged mice (Figure 8G). These results indicated that CCDC25 served as a key target through which NETs promoted skin photodamage.

## 4 Discussion

In this study, we identified abundant NETs infiltration in skin lesions of patients with actinic dermatitis. We established a UVB‐induced photodamaged model in mice, which showed significant NETs infiltration in the irradiated skin areas. NETs inhibition alleviated the skin damage. Additionally, CCDC25, expressed on keratinocytes, served as a key receptor for NETs‐mediated MAPK activation.

UVB radiation can cause skin damage directly or indirectly, accompanied by skin cells apoptosis, redox imbalance, inflammatory cytokine release, and DNA damage (Nichols and Katiyar 2010). Neutrophils, key responders in the immune system, are known to infiltrate skin abundantly and participate in the pathogenesis of skin photodamage upon UV exposure (Skopelja‐Gardner et al. 2021; Yano et al. 2005). NETs are web‐like structures generated by neutrophils, composed of extracellular chromatin modified by histones and numerous granular proteins (Yang et al. 2020). Recent studies have revealed their detrimental roles in both infectious and non‐infectious diseases (Castanheira and Kubes 2019; Fousert et al. 2020). Therefore, targeting NETs has emerged as a potential therapeutic strategy. GSK484, a novel PAD4 inhibitor, has been demonstrated potential in inflammatory diseases by inhibiting NETs generation and ameliorating disease severity (Hu, Hua, et al. 2023; Hu, Li, et al. 2023; Wang et al. 2023). Yano et al. (2005) observed neutrophil infiltration and abundant NE production in healthy human skin following UVB irradiation for two consecutive days. Using immunofluorescence staining (CitH3 and NE was used as NETs markers) combined with H&E staining, we identified significant neutrophil infiltration accompanied by NETs formation in sun‐associated dermatoses such as AK and CAD. Furthermore, previous studies indicate that wavelengths ranging from UVA (365 nm) to visible light (625 nm) can stimulate neutrophil NETosis (Arzumanyan et al. 2023). We confirmed that UVB also induced NETs formation in vivo and quantifying NETs via IF. What's more, in UVB‐induced acute photodamaged mice models, both traditional Chinese herbal medicine and DNase I were shown to promote skin lesions recovery and reduce inflammation by inhibiting NETs generation (Inaba et al. 2024). Our results also demonstrated that intraperitoneal injection of GSK484 during UVB irradiation mitigated skin photodamage in mice by suppressing NETs generation, ameliorating apoptosis, reducing inflammatory cytokine release, and improving redox imbalance.

To elucidate the mechanism by which NETs regulate skin photodamage, we performed RNA‐sequencing on the UV‐exposed mice group and the UV‐exposed intervention of GSK484 mice group. The MAPK pathway was enriched by KEGG enrichment analysis. The MAPK signaling cascade comprises p38, JNK, and ERK. Unexpectedly, following NETs inhibition in the intervention group, the activity of the p‐JNK/JNK pathway within the MAPK cascade was downregulated, while the activities of p‐p38/p38 and p‐ERK/ERK pathways remained unaffected. This result suggested that NETs likely promoted skin photodamage specifically through activation of the p‐JNK/JNK pathway within the MAPK signaling network.

CCDC25 is a receptor for NET‐DNA. NETs facilitate tumor metastasis by binding to CCDC25 on the surface of cancer cells (Yang et al. 2020). Zhu et al. found that in skin cells, CCDC25‐mediated activation of the PI3K/AKT pathway is participated in the promotion of fibroblast proliferation and migration by low concentrations of NETs (Chu et al. 2024). In our UVB‐induced mouse models, we observed increased CCDC25 protein levels in the skin of untreated photodamaged mice, while CCDC25 expression was reduced by NETs inhibition. Previous studies indicate that NETs can regulate cells via TLRs (Mi et al. 2023; Shao et al. 2019). After treating HaCaT cells with NETs extracted in vitro, we also detected increased mRNA expression of some TLRs, with CCDC25 exhibiting the most significant upregulation. Our research demonstrated that beyond exerting pro‐apoptotic and pro‐inflammatory effects in the photodamaged animal model, NETs intervention on HaCaT cells in vitro also induced apoptosis, increased inflammatory cytokines, and caused redox imbalance. Nevertheless, disrupting NETs structure using DNase I or silencing CCDC25 expression via siRNA abolished these NETs‐induced pro‐apoptotic, pro‐inflammatory, and redox‐imbalancing effects. To investigate the specific mechanism by which NETs act through CCDC25 on HaCaT cells, we combined RNA‐sequencing data and found that NETs treatment increased p‐JNK/JNK protein levels, while the activity of p‐JNK/JNK decreased after silencing CCDC25 expression. We constructed a skin photodamage model by injecting adeno‐associated virus into the dorsal skin of animals to silence CCDC25 expression, and found that CCDC25 knockdown alleviated skin photodamage and inhibited activation of the p‐JNK/JNK pathway. Our results indicated that NETs exerted effects on keratinocytes via the CCDC25/p‐JNK/JNK pathway, thereby exacerbating skin photodamage.

In conclusion, NETs can contribute to UVB‐induced skin photodamage. GSK484 can mitigate UVB‐induced skin photodamage in mice by inhibiting NETs generation. NETs exacerbate skin photodamage by promoting keratinocyte apoptosis and inflammatory cytokine release via the CCDC25/p‐JNK/JNK signaling pathway.

## Author Contributions

Yang Zou designed the experiments and drafted the manuscript. Aijun Chen provided research funding. Yang Zou, Junzhi Li, and Yuting Peng performed the experiments; Yuting Peng and Ruolin Li acquired and collected the data; Yang Zou and Junzhi Li analyzed and verified the data; Aijun Chen, Ruolin Li, and Rui Hu conceived the idea and edited the manuscript.

## Funding

This work was supported by the Chongqing Municipal Natural Science Foundation Innovative Development Joint Fund Project (CSTB2024NSCQ‐LZX0068).

## Ethics Statement

The Ethics Committee at The First Affiliated Hospital of Chongqing Medical University authorized this research, which adhered to the rules outlined in the Declaration of Helsinki. All experimental procedures were conducted in accordance with the National Institutes of Health Guide for the Care and Use of Laboratory Animals and approved by the Chongqing Medical University Animal Care and Use Committee.

## Conflicts of Interest

The authors declare no conflicts of interest.

## Supporting information


**Figure S1:** GSK484 suppresses CCDC25 expression in UVB‐induced photodamaged mouse skin.
**Figure S2:** PAD4 knockout mitigates UVB‐induced skin photodamage in mice.
**Figure S3:** Validation of CCDC25 knockdown efficiency by siRNA.
**Figure S4:** CCDC25 knockdown inhibits NETs‐induced inflammatory cytokines.
**Figure S5:** Validation of CCDC25 knockdown efficiency in skin by adeno‐associated virus.
**Table S1:** The primer sequences.

## Data Availability

All data are available from this article; additional data can be obtained by contacting the corresponding author.
