# Supplementary material for: Neutrophil Extracellular Traps Exacerbate UVB‐Induced Photodamage in HaCaT Cells and Mouse Skin via CCDC25/MAPK Pathway
Source: Aging Cell. 2026 Jul 23;25(8):e70640. doi: 10.1111/acel.70640 (PMC13395479; doi:10.1111/acel.70640)
Supplement: Supplementary file 1 — Figure S1: GSK484 suppresses CCDC25 expression in UVB‐induced photodamaged mouse skin. Figure S2: PAD4 knockout mitigates UVB‐induced skin photodamage in mice. Figure S3: Validation of CCDC25 knockdown efficiency by siRNA. Figure S4: CCDC25 knockdown inhibits NETs‐induced inflammatory cytokines. Figure S5: Validation of CCDC25 knockdown efficiency in skin by adeno‐associated virus. Table S1: The primer sequences. [file ACEL-25-e70640-s001.docx]

Supplementary figure.

Figure S1:





**A**

**B**


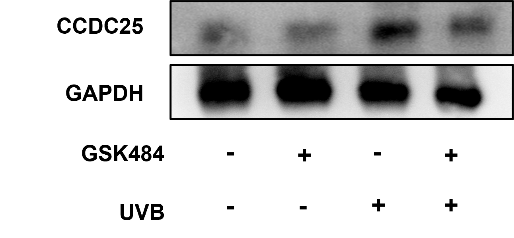


Figure S2:

**A**


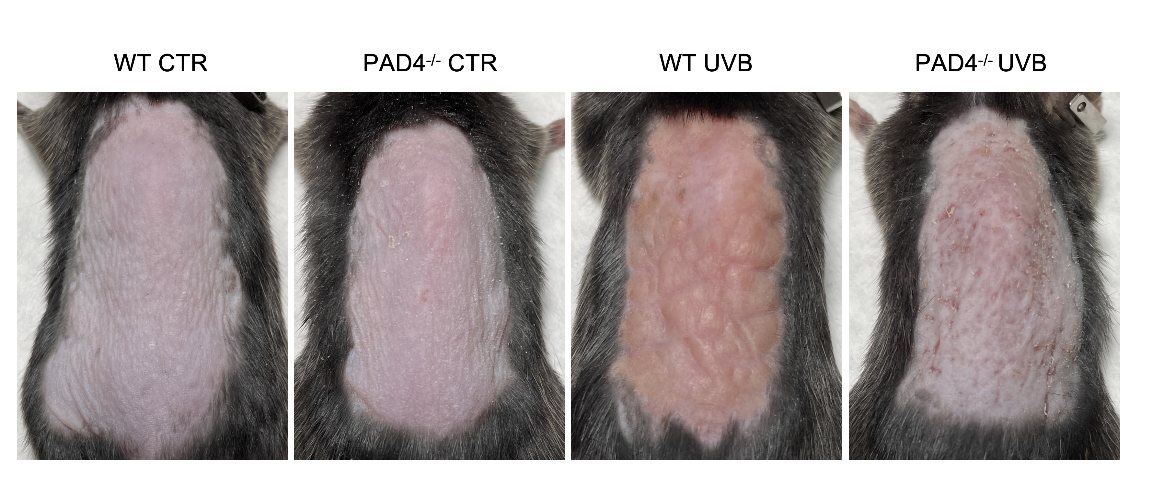


**B**


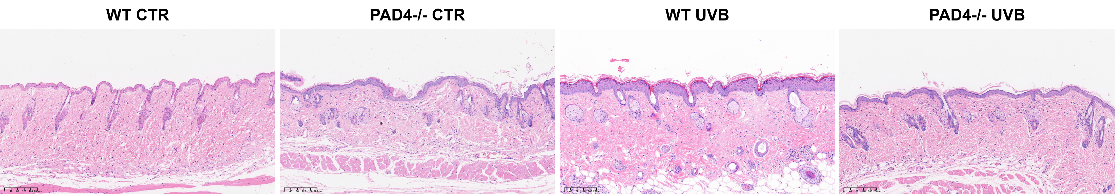


**C**





**D**







**E**


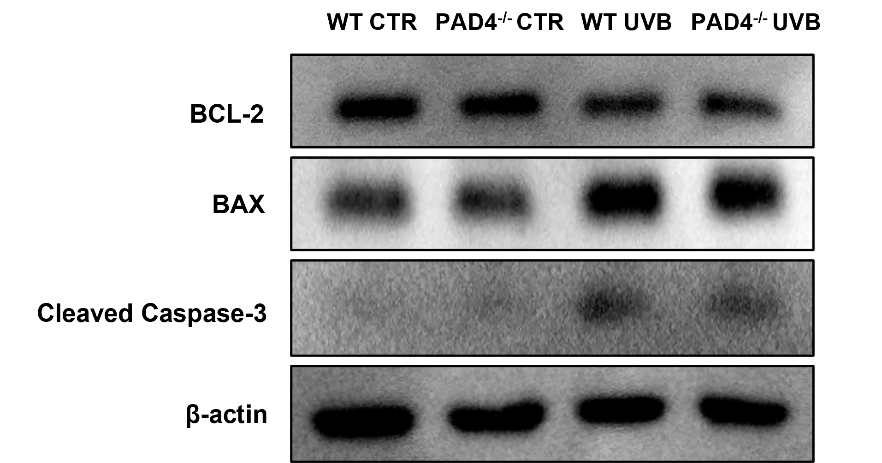


**F**









**G**






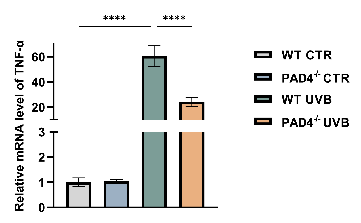


Figure S3：

**B**

**A**





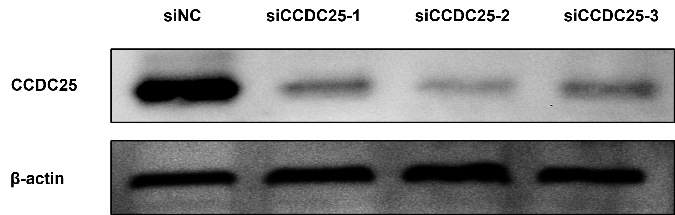


Figure S4:

**B**

**A**


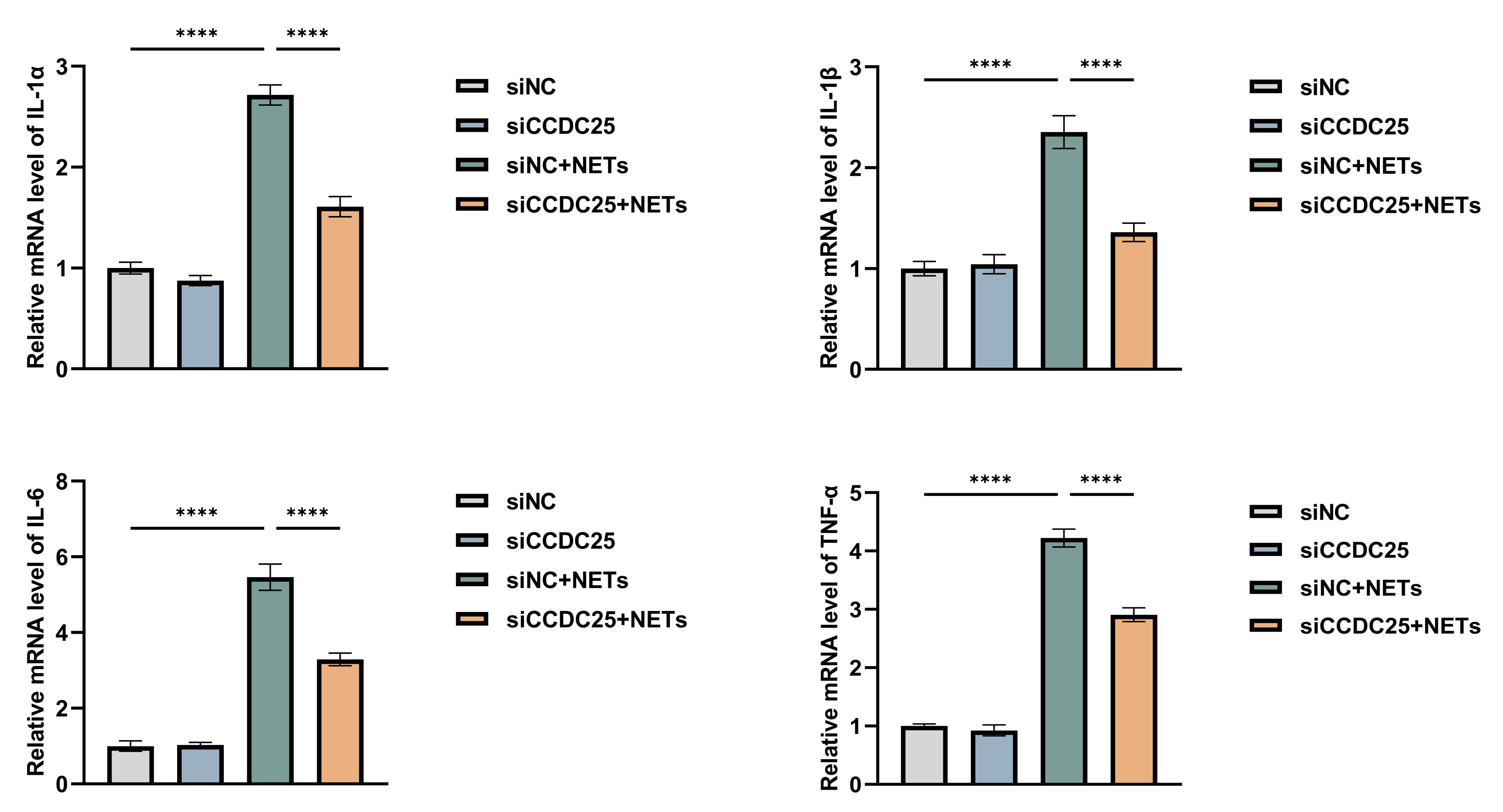


**D**

**C**

Figure S5:


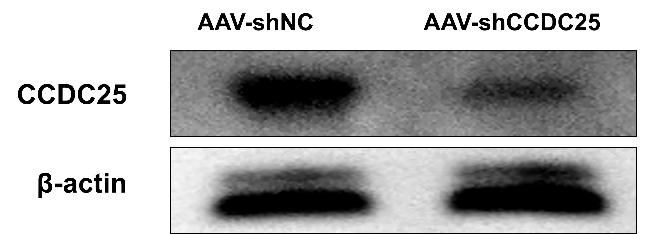


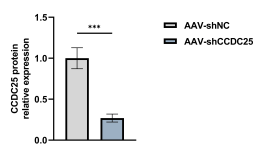


**B**

**A**

Figure legend：

Figure S1. GSK484 suppresses CCDC25 expression in UVB-induced photodamaged mouse skin. (A-B) CCDC25 protein levels in skin samples detected by Western blotting, with quantification performed by Image J software. The values are presented as the mean ± SD. *P < 0.05, **P < 0.01, ***p < 0.001. Each experimental procedure was independently replicated three times, each yielding similar results.

Figure S2. PAD4 knockout mitigates UVB-induced skin photodamage in mice. (A) PAD4 knockout ameliorates the skin appearance in UVB-exposed mice. (B) H&E staining of skin sections across experimental groups. Scale bar: 200 μm. (C) Quantification of the thickness of skin in the H&E staining. (D) SOD activity and MDA content in mouse skin samples. (E) Protein levels of Cleaved Caspase-3, BCL-2 and BAX in skin samples detected by Western Blotting. (F) Quantification of results shown in panel (E). (G) Relative mRNA levels of IL-1β, IL-6, and TNF-α in mouse skin samples. The values are presented as the mean ± SD. *P < 0.05, **P < 0.01, ***p < 0.001. Each experimental procedure was independently replicated three times, each yielding similar results.

Figure S3. Validation of CCDC25 knockdown efficiency by siRNA. (A-B) CCDC25 protein levels in cells detected by Western blotting, with quantification performed by ImageJ software.The values are presented as the mean ± SD. *P < 0.05, **P < 0.01, ***p* < 0.001. Each experimental procedure was independently replicated three times, each yielding similar results.

Figure S4. CCDC25 knockdown inhibits NETs-induced inflammatory cytokines. (A-D) Relative mRNA levels of IL-1α, IL-1β, TNF-α and IL-6 in NETs-stimulated HaCaT cells following CCDC25 knockdown. The values are presented as the mean ± SD. *P < 0.05, **P < 0.01, ***p < 0.001. Each experimental procedure was independently replicated three times, each yielding similar results..

Figure S5. Validation of CCDC25 Knockdown Efficiency in Skin by Adeno-Associated Virus. (A-B) CCDC25 protein levels in skin samples detected by Western Blotting, with quantification performed by ImageJ software. The values are presented as the mean ± SD. *P < 0.05, **P < 0.01, ***p < 0.001. Each experimental procedure was independently replicated three times, each yielding similar results.

Supplementary table 1.

The primer sequences.

| **Target genes** | **Sequence** |
| --- | --- |
| IL-1β (mouse) | Forward: AGACGGCTGAGTTTCAGTGAG   Reverse: AAGGTGCTGATCTGGGTTGG |
| IL-6 (mouse) | Forward: CCCCAAATTTCCCAAATGCTCTCC Reverse: GGATGGTCTTGGTCCTTAGCC |
| TNF-α (mouse) | Forward: ATGCCTCCTCTCTCCTCAGT Reverse: TTTGCTACGACGTGGGCTAC |
| Gm4610 (mouse) | Forward: GACAGGGTGGGGTAGCATTC Reverse: GGGCTGCGATGCTTATCTCT |
| Cd209f (mouse) | Forward: TGGAGAGCAGGGATTTGCTG Reverse: GGAGACTTGAACCAGGGTGG |
| Gm8210 (mouse) | Forward: GTCTGTCCGAATCTCCCCTT Reverse: CAATCCGTGCATTGTCCAGC |
| Oasl2 (mouse) | Forward: TGCCTGGGAGAGAATCGAGA Reverse: TGGAAATAGGTCCTTCACCACC |
| β-actin (mouse) | Forward: GCTCTGGCTCCTAGCACCAT  Reverse: GCCACCGATCCACACAGAGT |
| IL-1β | Forward: GAGCCGGGTGACAGTATCAG Reverse: TCTCACTGAAACTCAGCCGT |
| IL-6 | Forward: GAGAGTGATTGAGAGTGACCAC Reverse: CACCCTCTGCACCCAGTTT |
| TNF-α | Forward: CCTGTGAGGAGGACGAACAT Reverse: TTTGAGCAGAAAGAGGTTGAG |
| GAPDH | Forward: CTGGGCTACACTGAGCACC Reverse: AAGTGGTCGTTGAGGGCAATG |
